# The Public Health Legacy of Polio Eradication in Africa

**DOI:** 10.1093/infdis/jix034

**Published:** 2017-07-01

**Authors:** Allen S. Craig, Rustam Haydarov, Helena O’Malley, Michael Galway, Halima Dao, Ngashi Ngongo, Marie Therese Baranyikwa, Savita Naqvi, Nima S. Abid, Carol Pandak, Amy Edwards

**Affiliations:** 1 Polio Eradication Branch, Centers for Disease Control and Prevention, Atlanta, Georgia;; 2 Eastern and Southern Africa Regional Office, UNICEF, Nairobi, Kenya;; 3 African Regional Office, World Health Organization, Brazzaville, Republic of Congo;; 4 Bill and Melinda Gates Foundation, Seattle, Washington;; 5 Western and Central Africa Regional Office, UNICEF, Dakar, Senegal;; 6 UNICEF, N'djamena, Chad; 7 Eastern Mediterranean Regional Office, World Health Organization, Cairo, Egypt; and; 8 Rotary International, Evanston, Illinois

**Keywords:** Polio, Africa, transition, legacy.

## Abstract

The legacy of polio in Africa goes far beyond the tragedies of millions of children with permanent paralysis. It has a positive side, which includes the many well-trained polio staff who have vaccinated children, conducted surveillance, tested stool specimens in the laboratories, engaged with communities, and taken care of polio patients. This legacy also includes support for routine immunization services and vaccine introductions and campaigns for other diseases. As polio funding declines, it is time to take stock of the resources made available with polio funding in Africa and begin to find ways to keep some of the talented staff, infrastructure, and systems in place to work on new public health challenges. The partnerships that helped support polio eradication will need to consider funding to maintain and to strengthen routine immunization services and other maternal, neonatal, and child health programs in Africa that have benefitted from the polio eradication infrastructure.

The tragic impact of polio in Africa is borne by the many millions of disabled children and adults who have suffered from muscles weakened by the ravages of polio. However, there is a positive legacy of the years of polio eradication efforts in Africa—the African polio focal points, healthcare workers, surveillance officers, social mobilizers, laboratorians, and vaccinators who serve as a well-trained workforce prepared to fight other current and future public health battles. These dedicated staff have accomplished great things—not only in the fight against polio but also in improving health throughout the continent. They have enhanced routine immunization programs and perfected the use of mass vaccination campaigns against polio and other vaccine-preventable diseases (VPDs) to raise the immunity of large population groups, including nomads and persons living in insecure communities. They have emphasized the importance of having and using high-quality data for decision making. They have learned how to respond to a complex public health emergency using the Emergency Operations Center model. Awareness and trust were built as they engaged with the public, community and religious leaders, and traditional healers about healthy behaviors and the importance of childhood vaccinations. Finally, infectious disease surveillance was strengthened as laboratory expertise was decentralized and active searching for polio cases helped identify outbreaks [1].

The Global Polio Eradication Initiative (GPEI) and its partners invest substantial funds in the polio program in Africa. These funds are used to hire and train many public health workers and have been critical to the polio eradication progress seen thus far. Funds allocated for eradication in the highest-risk African countries are detailed in Table 1 [2]. Operational costs make up the largest proportion and include personnel, equipment, supplies, and transportation. The total for 2015 was US$466 million.

**Table 1.** External Funding Requirements in Polio-Endemic and Highest-Risk Countries, Excluding Program Support Costs—2015 (All Figures in US$ Millions)

Subregion and country	AFP surveillance	Social mobilization	Technical assistance	OPV	Operational costs	Total costs
West/Central Africa						
Nigeria	16.32	26.55	74.84	55.89	114.41	288.02
Chad	1.27	2.73	7.96	1.61	2.09	15.66
Cameroon	0.41	2.73	0.57	3.29	3.23	10.23
Niger	0.59	0.58	1.78	2.52	4.62	10.09
Mali	0.25	0.21	0.14	2.39	3.23	6.22
Burkina Faso	0.27	0.16	0.26	1.13	1.61	3.43
Benin	0.18	0.21	0.26	1.18	1.65	3.47
Guinea	0.18	0.15	0.09	1.07	1.51	2.99
Cote D'Ivoire	0.29	0.22	1.09	1.38	1.61	4.59
Central African Republic	0.47	0.71	0.63	0.44	0.76	3.01
Democratic Republic of Congo	2.25	2.45	11.43	0.91	3.63	20.67
Angola	1.9	0.13	10.08	0.3	0.07	12.48
Liberia	0.23	0.22	0.49	0.34	0.91	2.19
Gabon	0.09	0.3	0.28	0.1	0.2	0.97
Equatorial Guinea	0.04	0.18	0.15	0.05	0.3	0.72
Congo	0.14	0.28	0.34	0.37	0.73	1.86
Sierra Leone	0.23	0.37	0.44	0.52	1.07	2.63
Senegal	0.32	0.16	0.16	0.46	0.46	1.56
Horn of Africa						
Somalia	2.42	5.06	7.35	1.87	6.09	22.79
Ethiopia	3.06	1.58	3.19	2.32	6.66	16.81
Kenya	0.44	0.9	1.87	1.21	4.23	8.65
South Sudan	1.27	1.14	4.43	0.99	4.12	11.94
Sudan	1.27	0.23	0.62	0.79	1.82	4.72
Uganda	0.4	0.1	0.8	0.64	1.08	3.02
Yemen	0.19	0.43	0.19	1.45	2.09	4.35
Middle East						
Egypt	0.38	0.3	0.38	1.54	0.4	3
Total (percentage)	34.86 (8%)	48.08 (10%)	129.82 (28%)	84.76 (18%)	168.58 (36%)	466.07 (100%)

Abbreviations: AFP, acute flaccid paralysis; OPV, oral polio vaccine.

As the Lake Chad region mounts an aggressive response to the wild poliovirus resurgence identified in Nigeria after 2 years without a reported case in Africa, public health systems in many African countries face the specter of decreased polio funding, which currently helps bolster the entire continent’s public health and immunization systems. Because of the success of GPEI globally, funding decreased in 2016 and may continue to decrease through the end of 2019 and beyond (see Figure 1) [3]. The Global Polio Eradication Initiative has generous donors who contribute substantially to the current effort to eradicate wild poliovirus transmission, stop outbreaks of circulating vaccine-derived poliovirus, enhance polio surveillance, and improve polio vaccination programs worldwide (Table 2) [2]. National governments and their international supporters need to decide whether resources that were allocated for polio eradication can now start to be redirected to other essential maternal, neonatal, and child health (MNCH) programs, in particular routine immunization and surveillance [1, 4].

**Figure 1.**
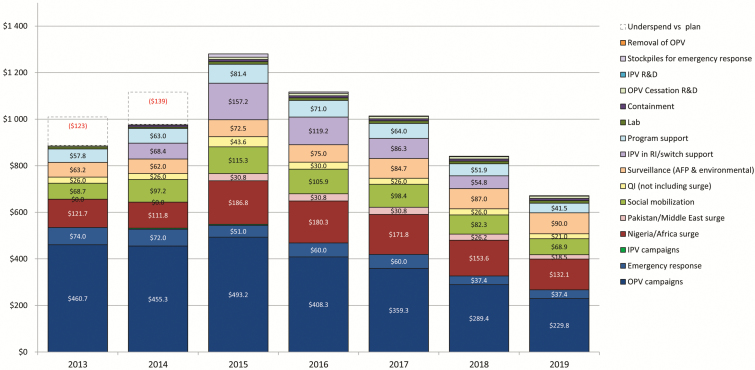
Estimated costs for polio eradication 2013–2019, by activity ($US millions, not including India self-funded costs). Abbrevations: AFP, acute flaccid paralysis; IPV, inactivated polio vaccine; OPV, oral polio vaccine; QI, quality improvement; R&D, research and development; RI, routine immunization.

**Table 2.** Summary of Confirmed Funding Against Global Vaccine Summit Commitments (All Figures in US$ Millions)

G7 and European Commission	Committed funding at the April 2013 Vaccine Summit^k^	Confirmed funding against the Global Polio Eradication Initiative financial resource requirements as of 1 April 2016
Canada^a^	243.53	197.97
European Commission	6.5	27.06
Germany^b^	151.7	111.67
Japan^c^	9.7	43.5
United Kingdom^d^	457	480.67
USA^e^	90.6	401.84
Non-G7 Office for Economic Development and Coorperation		
Australia^f^	34.55	55.24
Finland	0.53	0.53
Ireland	6.5	6.36
Luxembourg	0.7	3.21
Norway^g^	252.45	211.93
Other donor countries		
Brunei Darussalam	0.05	0.05
Isle of Man	0.14	0.05
Liechtenstein	0.02	0.08
Monaco	0.35	0.95
Saudi Arabia	15	12.35
Private sector/Nongovernmental donors		
Al Ansari Exchange	1	1
Abu Dhabi-Crown Prince^h^	120	53.49
Bill & Melinda Gates Foundation^i^	1 800.00	1 108.39
Korean Foundation for International Healthcare/Community Chest of Korea	1	3
Private philanthropists/high net worth individuals	335	89.68
Rotary International^i^	76.81	344.53
United Nations Foundation	0.75	0.8
Multilateral sector		
The Vaccine Alliance (Gavi)/International Finance Facility for Immunization	24	25.21
Islamic Development Bank/Government of Pakistan	227	225.46
United Nations Children’s Fund	64.5	56.08
World Bank (Grant to Afghanistan)	10	11
World Bank Investment Partnership, Bank Portion	50	50
World Health Organization	4.27	12.77
Domestic resources		
Angola	7.3	6.54
Bangladesh	10	10
Nepal	0.9	0.67
Nigeria^j^	40	239.37
TOTAL	4 041.85	3 791.45

^a^Canada pledged Can$ 250 million for the 2013–2018 period. Canada also provided an additional Can$3 million for the 2013–2014 Horn of Africa outbreak. Contributions do not include approximately Can$28.6 million for activities in Pakistan, Nigeria, and Ukraine that lie outside the Global Polio Eradication Initiative (GPEI) budget but support the overall goal of polio eradication.

^b^Germany also provided more than €13 million in 2013–2014 for the Middle East outbreak in addition to current disbursements under its €105 million 2013–2017 pledge.

^c^Since 2011, Japan has supplemented its traditional grant financing with innovative financing in partnership with the Bill & Melinda Gates Foundation. Under this loan conversion model, Japan has provided development assistance loans to Nigeria (approximately US$70 million, 2015–2016) for vaccine and operational costs. If performance criteria are met, the BMGF will repay the loan credit to the Japan International Cooperation Agency on behalf of the Nigerian government, in effect converting the loan to a grant.

^d^The United Kingdom committed £300 million to polio eradication for the 2013–2018 period, comprised of “core” and “match” funds. The figures for 2016–2018 include £28 million in “match” funds as well as £27 million to Gavi for inactivated polio vaccine (IPV) procurement. The United Kingdom also provided an additional £13.8 million for the 2013–2014 Horn of Africa and Middle East outbreaks.

^e^United States figures reflect the actual amount received directly by the 2 implementing agencies, consistent with the United Nations revenue recognition policy. The fiscal year 2016 Congressional allocation is US$228 million. The 2016 figure represents disbursements to WHO and UNICEF from the US Centers for Disease Control and Prevention and United States Agency for International Development against the GPEI’s 2016 budget to date.

^f^Australia’s figures include funding received for the 2013–2015 period under 2 commitments: 2011–2015 (Aus$50 million) and 2015–2019 (Aus$36 million).

^g^Norway’s figures reflect all confirmed funding to Gavi (2013–2019) and funding to WHO (2013–2015).

^h^Abu Dhabi-Crown Prince figures include funds via the UAE Pakistan Assistance Programme.

^i^In 2013, Rotary pledged up to US$175 million for the 2013–2018 period, which will be matched by the Bill & Melinda Gates Foundation. Contributions under this scheme are included in Rotary’s figures. Rotary’s contributions to the GPEI are through the Rotary Foundation.

^j^Nigeria’s figures include domestic resources from loans from the World Bank (US$85.6 million) and the Japan International Cooperation Agency loan conversion (US$70.3 million).

^k^Only includes donors who pledged funds at the Vaccine Summit. See www.polioeradication.org/financing.aspx for additional information on contributions, including those that are not against the GPEI budget (non-Financial Resource Requirement report).

This article summarizes areas where polio funding has made an impact on public health in Africa and highlights where continued funding is needed. When smallpox eradication finally succeeded in Africa, the program helped launch the Expanded Programme on Immunizations (EPI) [5]. Now, in a new century with continuing public health needs in Africa, it is critical that national governments and the global public health community help transition polio-funded immunization and public health programs to new funding so that the staff and expertise developed under GPEI are not lost.

## USE OF POLIO ASSETS FOR ROUTINE IMMUNIZATION

It is not straightforward to isolate GPEI’s impact on routine immunization services, but it is clear that polio eradication–funded assets have supported systematic infant immunization, particularly in countries with the most polio eradication investments. The Boston Consulting Group survey [6] commissioned by GPEI in the 10 countries where the majority of polio program staff and resources are located showed that on average polio frontline workers spent 22% of their time on routine immunization activities. When adding other vaccination campaigns, new vaccine introductions, and MNCH activities, the survey found that polio-funded staff were spending about 50% of their time on nonpolio programs. Lower frequency of campaigns, strong government commitment, and a high level of transparency and effective accountability mechanisms were mentioned as critical to helping countries allocate staff time to routine immunization and other programs.

The Global Polio Eradication and Endgame Strategic Plan 2013–2018 places additional emphasis on strengthening routine immunization services [7]. Gavi, the Vaccine Alliance, partnered with GPEI in 2014 to support the introduction of inactivated polio vaccine (IPV) in the routine immunization schedule. The terms of reference of polio-funded personnel in several countries reflect work on routine immunization services. Funding support for routine immunization services between campaign activities has been provided through GPEI to countries that had the most polio assets, and some countries extended support to include other interventions, such as follow-up of dropouts from routine immunization, birth registration, and screening for malnutrition. Additionally, such funding allowed the targeting of the lowest performing areas in high-risk districts in Chad, the Democratic Republic of Congo, and Nigeria in 2014–2016.

Nigeria and Central African Republic have used polio campaigns to deliver an integrated health package that includes other routine vaccines. Many countries also deliver vitamin A and deworming during polio campaigns, with some areas extending this to include bed net distribution and promotion of handwashing. These “pluses,” as they are often called, help both encourage parents to have their child vaccinated and accomplish the distribution of other potentially lifesaving interventions. Polio campaigns have also provided a platform for vaccinations and other health interventions in hard-to-reach groups in remote and insecure areas.

The Global Polio Eradication Initiative has a number of capacities that countries may leverage to strengthen routine immunization systems and improve coverage and equity. These include supply chain capacity; coordination and accountability; microplanning, including mapping of missed children and special populations; demand generation and engaging with communities; and working in areas of insecurity.

As more detailed asset mapping of GPEI-funded assets becomes available in each country, this will inform the conversation on government-led transition planning. Funding from national governments, partners, and Gavi will be critical to allow countries to utilize GPEI staff and expertise to sustainably support routine immunization in the years after polio eradication.

## POLIO LEGACY AND HEALTH SYSTEMS: A TRANSITION FROM SUPPORT TO STRENGTHENING

The Global Polio Eradication Initiative has made significant investments in health systems to help stop poliovirus transmission. Mapping of polio assets identified specific initiatives to improve leadership and governance, empower communities to engage and participate in polio and health issues, and strengthen information systems to facilitate the availability and use of quality data to guide local actions. However, the question remains whether GPEI has contributed to health system strengthening—defined as interventions that foster lasting changes to the main performance drivers of a functional health system [8, 9]. Polio eradication intervention was considered short-term health system support that would not last beyond polio funding.

In Chad, Republic of Congo, Democratic Republic of Congo, and Nigeria, polio-funded staff were given a broader mandate—to strengthen the human resource capacity of routine immunization programs. In Cameroon, Republic of Congo, and Democratic Republic of Congo, new public–private partnerships used mobile phone–based education to support supplementary polio immunization activities, which opened new horizons for the use of innovative technologies in creating demand and increasing social accountability for MNCH in general and routine immunizations in particular. In Chad, Democratic Republic of Congo, and Guinea, the use of lot quality assurance sampling in monitoring the performance of polio campaigns has brought in a new tool to strengthen data availability and quality in settings with weak health information systems.

Countries have started using these promising polio eradication practices to strengthen health systems in MNCH programs beyond polio, including routine immunizations. Chad is 1 of the 12 countries accounting for 70% of unimmunized children globally. Only 50% of children receive 3 doses of polio vaccine, 25% are completely vaccinated, and 19% receive no vaccine doses [10]. To improve this situation, United Nations Children’s Fund (UNICEF) supported the Chadian Ministry of Health (MoH) to introduce a community register in 2015 using polio funds from the Bill and Melinda Gates Foundation (BMGF). The register was implemented through community health workers to identify the children missing vaccinations, who constitute the group most vulnerable to polio outbreaks. Preliminary data from 10 districts with the register showed that during the implementation period the number of children aged 0–23 months partially or completely vaccinated increased from 17167 to 94555 and from 9893 to 71015, respectively. The children included in this program benefited by improved polio and routine immunizations. Learning from this early experience in Chad, the community register is being introduced in Guinea and Republic of Congo using nonpolio funds as a polio transition health system strengthening intervention.

The ongoing polio transition planning process provides an opportunity for countries to shift polio-funded health system support interventions toward health system strengthening using domestic and other sustainable funding opportunities. This polio transition effort, combined with Gavi health systems strengthening support, will give African countries an opportunity to make much-needed improvements in their health systems.

## SURVEILLANCE

Acute flaccid paralysis (AFP) surveillance is 1 of the 4 pillars of GPEI; the others are routine immunization, supplementary immunization activities, and targeted “mop up” campaigns. The key steps in AFP surveillance are (1) finding and reporting children with AFP, (2) transporting stool samples for laboratory analysis, and (3) isolating and sequencing polioviruses, if present. An environmental surveillance system (testing sewage for polioviruses) is also operating in 11 African countries to supplement AFP surveillance [11]. Polio surveillance in Africa involves hundreds of World Health Organization (WHO) and MoH staff who regularly search for AFP cases in health facilities, during meetings with healthcare workers, traditional healers, and community health workers, and in hundreds of sewage samples collected each month. Although still in need of improvement, the AFP surveillance system is functioning at some level in all high-risk African countries [12].

Stool and sewage samples collected through the surveillance network are tested in WHO-accredited laboratories within the Global Polio Laboratory Network (GPLN). The GPLN consists of 145 laboratories in a 3-tiered structure—national laboratories, regional reference laboratories, and global specialized laboratories. All laboratories are continually monitored for their adherence to quality indicators and have annual accreditation assessments [13].

Certification of a WHO region as polio-free requires at least 3 years of timely and sensitive surveillance [14]. Each country in Africa presents its surveillance and vaccination data to the respective Regional Certification Commissions until all countries have adequately documented their polio-free status.

Innovations in surveillance are often needed because one approach does not fit all settings. In Somalia, an estimated 540786 (9%) and 1665027 (27%) of a total 5999444 children aged <15 years in the country live in inaccessible and partially accessible areas, respectively. During the 2013–2014 outbreak in Somalia, the polio program recruited and trained 635 village polio volunteers (VPVs) to identify and report AFP cases and collect stool samples. As of 12 June 2016, VPVs reported 75% of AFP cases in the inaccessible areas and 43% in the partially accessible areas. The cases were investigated in a timely manner, and the reverse cold chain was well maintained, as reflected by 100% and 93% of AFP cases being investigated within 48 hours and a nonpolio enterovirus (NPEV) isolation rate of 31% and 9% in the inaccessible and partially accessible areas, respectively [15]. As GPEI transitions to a polio-free world, surveillance programs like the one in Somalia need to be maintained and used for polio and other diseases of public health importance such as measles.

Polio assets include thousands of trained surveillance officers, WHO-accredited laboratory network staff and resources (including 20 polio laboratories in Africa), and surveillance procedures and lessons learned [1]. Polio surveillance officers and the vast GPLN can be redesigned to support the detection and laboratory diagnosis of other diseases. Lessons learned from polio surveillance can also support the Integrated Disease Surveillance System for communicable diseases by adopting innovative community-based approaches. This surveillance infrastructure can be used to conduct surveillance for other vaccine-preventable diseases such as measles, meningitis, and yellow fever, as well as diarrheal and unexplained respiratory illnesses.

## SOCIAL MOBILIZATION AND COMMUNICATION

Global Polio Eradication Initiative investments have created substantial communication assets over the years across Africa, which include social mobilization networks and multimedia platforms. Thousands of social mobilizers in Somalia, Ethiopia, South Sudan, Nigeria, Democratic Republic of Congo, Chad, and Cameroon are deployed to help reach every last child with the polio vaccine.

Typically, trained community mobilizers and their supervisors constitute multitiered networks that are deployed in areas at high risk for polio. They raise awareness and educate parents about polio vaccination campaigns, engage communities, build relationships with religious leaders to garner their support, and create social acceptance of polio vaccination. Equipped with health education tools and interpersonal communication skills, 1078 social mobilizers in South Sudan, 3616 in Somalia, 14000 in Nigeria, 14467 in Cameroon, and >30000 in Democratic Republic of Congo conducted door-to-door visits and community group meetings aimed at reducing missed children. In Nigeria these networks are deployed on a full-time basis, and in other countries like Democratic Republic of Congo they are mobilized at the time of polio campaigns. They helped develop community profiles and social map and collect social data from sites such as schools, daycare centers, places of religious congregation, transit points, and playgrounds to enlist influencers and reduce noncompliance [16].

Social mobilizers work during polio campaign days, but as part of the polio endgame strategy, they have also been active in accelerating routine immunization, reducing maternal and child deaths, and addressing malnutrition. Beyond development programs, these networks, which include groups of mothers, have demonstrated added value in emergency response—cholera and measles outbreaks in Democratic Republic of Congo, Cameroon, Chad, Somalia, South Sudan and drought in Ethiopia. In conflict areas, such as the Lake Chad region, they help displaced populations engage with camp dwellers and link them to social services. When Ebola cases were confirmed in Abuja in July 2014, polio community engagement networks were repurposed to actively find cases and track potential chains of transmission, helping Nigeria stop Ebola in 3 months [17, 18].

As GPEI resources scale down, investments made in developing efficient community mobilization and health promotion platforms can be redirected to other public health priorities. For example, in Somalia, the plan is for these networks to be supported by Gavi health system strengthening; in Democratic Republic of Congo discussions are ongoing with the MoH to absorb a critical mass of community mobilizers as part of the new National Community Health Policy; and Cameroon is currently working toward an agreement with non-GPEI partners to absorb polio social mobilizers.

## EMERGENCY OPERATIONS CENTERS

Beginning in 2012, GPEI began establishing emergency management and control facilities in the 3 remaining polio-endemic countries. National and subnational Emergency Operations Centers (EOCs) were opened in Nigeria (8 during 2012–14), Pakistan (6 during 2014–15), and Afghanistan (4 in 2016). All remain in operation and provide an important framework for a heightened command-and-control response to complex public health emergencies.

The EOCs addressed a particular problem in the polio program where management of program operations was spread across multiple institutions—government, United Nation agencies, and nongovernmental organizations—with each operating in relative isolation. The EOCs provide for the physical colocation of program resources under government leadership and are equipped with high-speed internet, printers, and computers for producing the analyses needed in real time to drive program decision making.

Government MoHs provide a strong leadership team, creating the opportunity to strengthen accountability of health workers and managers for polio vaccination activities. The United States Centers for Disease Control and Prevention (CDC) provides epidemiological and virologic input to the EOCs to help identify areas at highest risk for sustaining transmission and supports field operations. UNICEF’s experts address social issues impacting the program, including misinformation, mistrust, and refusal to vaccinate, as well as vaccine management. WHO focuses on surveillance and operations and provides the backbone to the program response. BMGF and Rotary International provide funding and staff. Within each EOC, the government and its implementing partners have created teams organized along thematic lines, including strategy, operations, logistics, communication and advocacy, and data management. Once the EOC begins tracking performance of operations teams in campaigns and social mobilization, the EOC has the authority to sanction poor-performing field staff.

Data-driven assessment of polio vaccination performance and local community health agency support before and during each campaign enables the EOC to shift focus to the highest-risk clusters and issues. Improved data collection from the field and use of satellite imagery allow the EOC to adapt new approaches rapidly and to experiment with interventions to solve complex vaccination and surveillance problems.

Vigorous program management allowed the EOC to identify issues and intervene to mitigate specific challenges, such as reaching children trapped by the ongoing Boko Haram insurgency in northeast Nigeria. Examples include “hit and run campaigns” where vaccinators enter a village in a large group during periods of relative calm to rapidly immunize as many children as possible and “firewalling” Boko Haram–controlled districts by establishing intensive immunization activities in adjacent, government-controlled areas to increase immunity in areas surrounding inaccessible districts.

Recent history provides evidence of the wider application of EOC processes and systems. From March to October 2014, the EOC structure in Nigeria was successfully leveraged for the response to the Ebola crisis. The rapid containment of Ebola transmission in Nigeria provided clear evidence of the wider application of the polio EOC model, a potent reminder of the legacy of the national polio program [19].

The key lessons learned from the polio experience of EOCs—rapid synthesis of data to drive program choices, heightened accountability around action in the field, and the assembly of multifunctional teams to tackle priority issues in real time—all have meaningful application for outbreak and emergency response and for delivering critical health services to families and children in Africa.

## WORKING IN COMPLEX PARTNERSHIPS

The Global Polio Eradication Initiative is a robust, multilateral, public–private partnership established to specifically attain the target of a polio-free world. The core global partners include WHO, UNICEF, Rotary International, BMGF, and the CDC. In Africa, this partnership has matured over time; the 5 core GPEI partners have collaborated in each endemic and outbreak country to ensure that WHO, UNICEF, MoHs, and local government are well supported. As Africa works toward becoming a polio-free continent, it is critical that the major partners, agencies, and donors continue to work well together to ensure a smooth transition into the postpolio era.

The Global Polio Eradication Initiative operates within a broad framework of intergovernmental and interagency cooperation and participation at the global, regional, national, subnational, and district level. At the country level, Interagency Coordinating Committees are in place to improve coordination among partners in support of immunization programs—to include polio eradication activities. Crucial elements of an effective Interagency Coordinating Committee that harnesses a strong partnership include leadership and active participation from a high authority within the MoH, a coordinated work plan among all country-level stakeholders, coordinated implementation, and collective monitoring and evaluation of program performance.

In security-compromised zones, GPEI partnered with a wide variety of stakeholders to negotiate access to conduct polio eradication activities. These partners included religious and community leaders, tribal elders, national and provincial governments (including the military and law enforcement authorities), and nonstate armed groups. Such partnerships allowed the program to proceed with conducting planned polio vaccination campaigns.

The GPEI partnership finds itself at a crucial point in history. Once the eradication goal is achieved and the GPEI partnership ends, strengthening existing partnerships and creating new ones will be key to continuing to improve access to immunizations in Africa. Examples of such partnerships include cooperation among governments, investment institutions, charitable foundations, the private sector, and regional organizations. Governments, donors, and communities should begin to think about new partnerships and continue to build on the strong partnerships created to eradicate polio.

## FUNDING THE TRANSITION AND BEYOND

The 54 countries in Africa rely to varying degrees on funds from donors to GPEI to support both polio immunization and other public health programs. In addition to helping fight polio, these funds have built capacity in health systems across the region critical for maintaining population immunity, as outlined in Boston Consulting Group surveys conducted in 2014–2015 [6]. With GPEI poised to ramp down its funding for polio eradication, the need to identify funding to maintain this capacity has taken on increased significance.

The process is designed to be led by national governments through mapping exercises that help to detail key assets and determine priorities for the future. Once these priorities are established, conversations with donors can begin, as budget cycles and appropriation processes must be considered. In several of the 16 African countries with the most GPEI funding, such as Sudan, Democratic Republic of Congo, and Chad, government leaders and partners have developed work plans to identify key polio assets, such as human resources, surveillance, and laboratory facilities, and have created budget projections for transitioning those critical resources. However, despite the completion of mapping exercises and the development of strategic plans, support from the global level for posteradication public health funding has not been secured. The Global Polio Eradication Initiative has provided tools to help guide the process [20], but in the face of many competing challenges and the ongoing need for polio eradication activities, this process has been slow to take off across Africa. Current stakeholders and key partners like Rotary International and BMGF serve as important advocates for moving the process forward, but governments must take ownership and make a case for continued support.

The Africa Health Strategy 2007–2015 points out that, although Africa has 10% of the world population, it bears 25% of the global disease burden and only 3% of the global health workforce [21]. Current programmatic sources such as Gavi, EPI, global health security, and pandemic preparedness funding have a role, but more needs to be done to cultivate a range of sources. Along with identifying global partners, countries will need to focus on prioritizing their own needs, creating program efficiencies, and developing innovative plans for ensuring the continuation of key functions during the transition process. A February 2016 report from the Center for Strategic and International Studies (CSIS) Global Polio Health Center highlights Ethiopia as an example where the US government contributes significantly to immunization coverage and disease surveillance in border populations. It urges the US government to support transition planning efforts in “Ethiopia and elsewhere as a way to fully leverage US investments in global polio eradication and to further US global health aspirations” [22].

The polio eradication program in Africa serves as an example of how collaborative efforts can impact the quality and reach of public health systems. This same collaborative effort among African countries and international donors has the potential to ensure that the resources built through polio eradication are effectively leveraged to benefit routine immunization services and other health priorities once polio has been eradicated. The timeframe to identify funding sources is short as polio budgets ramp down and the region faces the risk of losing the health gains made through the support of the polio eradication effort.

## CONCLUSIONS

Polio and other public health programs in Africa are at a crossroads. With progress in improving population immunity, sustaining a sensitive surveillance system, and strengthening outbreak response, Africa is moving closer to being polio-free. As a result, GPEI’s budget requirements are being systematically reduced in a manner to ensure the polio eradication gains to date are maintained and that the program globally remains on course to achieve eradication. The progress toward polio eradication invariably raises questions on how to best transition polio assets—people, infrastructure, processes, and knowledge—to other public health priorities as they are identified by national governments. This is of particular importance in Africa given its high child mortality and morbidity rates and the large investment by GPEI in several African countries. The transition planning underway provides an important opportunity to examine best practices and lessons learned, identify new possibilities for how polio assets can be used to support other public health priorities in the Africa region, and reinforce the unique partnerships that will help provide funding to strengthen routine immunization services and other maternal, neonatal, and child health programs.

## Notes

***Financial support.*** This work was supported by the Centers for Disease Control and Prevention.

***Supplement sponsorship.*** This work is part of a supplement coordinated by the Task Force for Global Health with funding provided by The Bill and Melinda Gates Foundation and the Centers for Disease Control and Prevention.

*Potential conflicts of interest.* All authors: No reported conflicts. All authors have submitted the ICMJE Form for Disclosure of Potential Conflicts of Interest. Conflicts that the editors consider relevant to the content of the manuscript have been disclosed.
